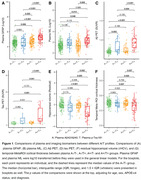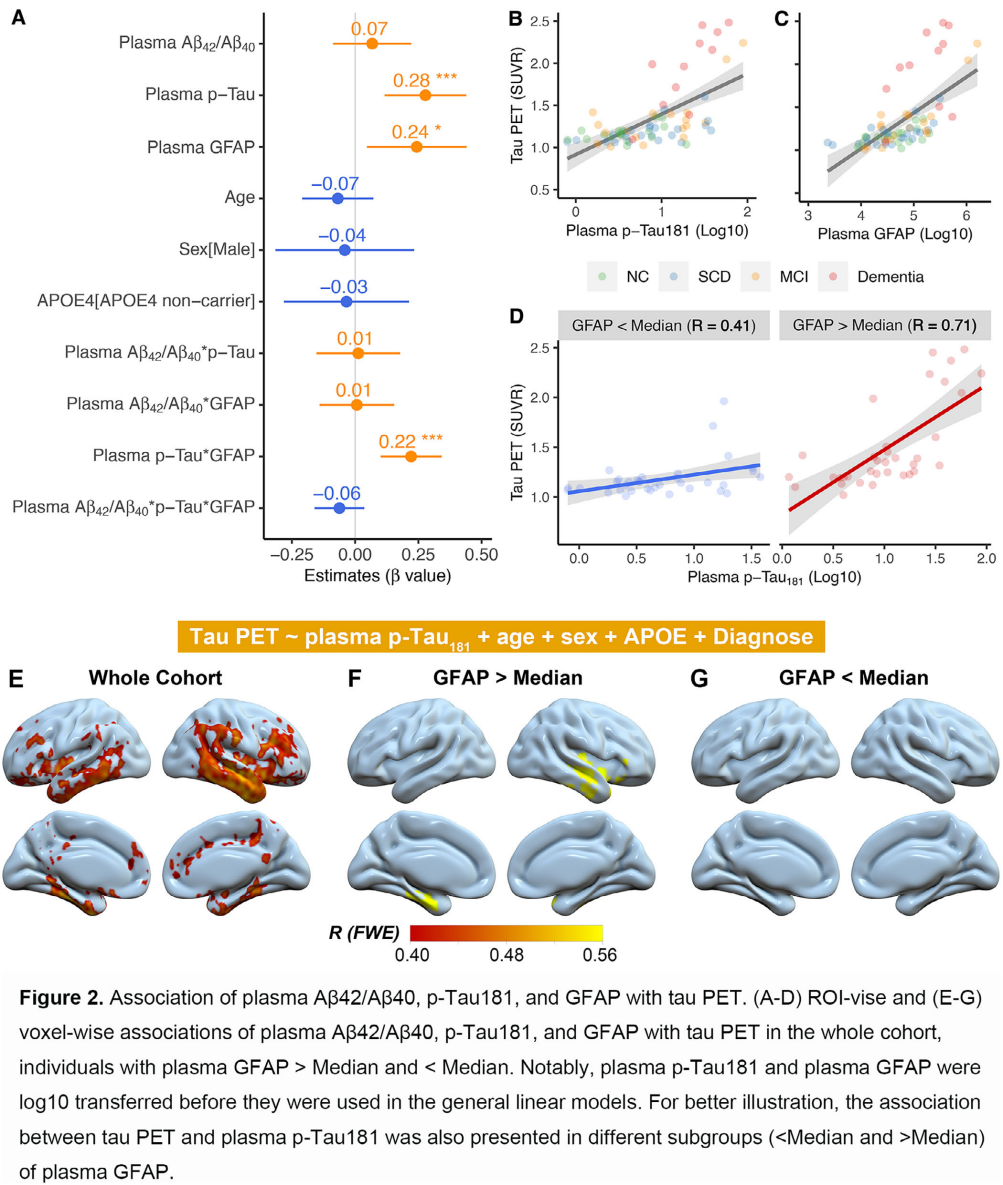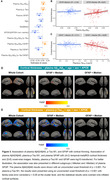# Plasma GFAP influences tau aggregation and cortical thickness thinning in Alzheimer’s disease

**DOI:** 10.1002/alz.091316

**Published:** 2025-01-09

**Authors:** Tengfei Guo, Anqi Li, Pan Sun, Zhengbo He, Yue Cai, Guoyu Lan, Dai Shi, Jie Lu, Ying Han

**Affiliations:** ^1^ Institute of Biomedical Engineering, Shenzhen Bay Laboratory, Shenzhen China; ^2^ Institute of Biomedical Engineering, Peking University Shenzhen Graduate School, Shenzhen China; ^3^ Institute of Biomedical Engineering, Shenzhen Bay Laboratory, Shenzhen, Guangdong China; ^4^ The Seventh Affiliated Hospital, Sun Yat‐sen University, Shenzhen, Guangdong China; ^5^ Department of Radiology, Department of Nuclear Medicine, Xuanwu Hospital, Capital Medical University, Beijing, Beijing China; ^6^ Department of Neurology, Xuanwu Hospital of Capital Medical University, Beijing, Beijing China

## Abstract

**Background:**

The thresholds of plasma β‐amyloid(Aβ)_42_/Aβ_40_ and phosphorylated Tau_181_ (p‐Tau_181_) to detect Alzheimer’s disease (AD) pathophysiology in Chinese community older adults have not been well established. Besides, it is still unclear about the role of astrocyte reactivity in Aβ plaque deposition, tau tangle aggregation, and neurodegeneration in AD.

**Method:**

In this study, we analyzed plasma Aβ_42_/Aβ_40_, p‐Tau_181_, glial fibrillary acidic protein (GFAP), and neurofilament light (NfL) using the Simoa platform in 470 Chinese older participants. Among them, 301, 195, and 70 individuals had MRI, Aβ PET, and tau PET images. The receiver operating characteristic curve analysis (Youden index) was conducted to define plasma Aβ_42_/Aβ_40_ (A) and p‐Tau_181_ (T) thresholds as £0.0609 and ³2.418 by comparing Aβ‐PET negative cognitively individuals and Aβ‐PET positive cognitively impaired patients. We compared plasma GFAP, Aβ plaque, tau tangle, plasma NfL, hippocampal volume, and temporal‐metaROI cortical thickness between different plasma A/T profiles and studied their association with each other using general linear models, controlling for age, sex, APOE‐ε4, and clinical diagnosis.

**Result:**

We found that plasma A+/T+ individuals had the most astrocyte reactivity, Aβ plaque, tau tangle, axonal degeneration, hippocampal atrophy, and temporal‐metaROI cortical thinning (Figure 1). Lower plasma Aβ_42_/Aβ_40_ (p < 0.001) and higher plasma p‐Tau_181_ concentrations (p < 0.001) were associated with higher Aβ PET SUVR (Figure 2). Besides, higher plasma p‐Tau_181_ (p < 0.001) and plasma GFAP (p = 0.015) concentrations were associated with higher temporal‐metaROI tau PET SUVR, and they also showed significant interactive relation (p < 0.001) with higher tau PET SUVR (Figure 2). Higher plasma GFAP (p = 0.012) was related to more shrinking in temporal‐metaROI cortical thickness and showed significant interaction with plasma Aβ_42_/Aβ_40_ (p = 0.042) and plasma p‐Tau_181_ (p < 0.001) at predicting temporal‐metaROI cortical thinning (Figure 3). The voxel‐wise imaging analysis replicated these findings.

**Conclusion:**

These findings offer significant reference for using plasma biomarkers to detect AD in the Chinese community older population and provide novel insights into understanding how astrocyte reactivity affects cortical tau aggregation and thickness thinning in AD. This study emphasizes the importance of controlling reactive astrogliosis to prevent AD progression.